# Soft-Templating of Sulfur and Iron Dual-Doped Mesoporous Carbons: Lead Adsorption in Mixtures

**DOI:** 10.3390/molecules25020403

**Published:** 2020-01-18

**Authors:** Dipendu Saha, Connelly P. Richards, Robert G. Haines, Nicholas D. D’Alessandro, Madeleine J. Kienbaum, Christian A. Griffaton

**Affiliations:** Department of Chemical Engineering, One University Place, Chester, PA 19013, USA; cprichards@widener.edu (C.P.R.); rghaines@widener.edu (R.G.H.); ndalessandro@widener.edu (N.D.D.); mjkienbaum@widener.edu (M.J.K.); cagriffaton@widener.edu (C.A.G.)

**Keywords:** mesoporous carbons, doping, adsorption, water pollution, lead

## Abstract

Lead pollution in drinking water is one of the most common problems worldwide. In this research, sulfur and iron dual-doped mesoporous carbons are synthesized by soft-templating with sulfur content 4.4–6.1 atom% and iron content 7.8–9 atom%. Sulfur functionalities of the carbons are expected to enhance the affinity of the carbon toward lead whereas iron content is expected to separate the carbon from water owing to its magnetic properties. All the carbons were characterized by pore textural properties, x-ray photoelectron spectroscopy (XPS), scanning electron microscopy (SEM) and energy dispersive x-ray (EDX). In order to study the Pb(II) removal efficiently of this carbon in competitive mode and to mimic the real-world use, one additional heavy-metal, including Cr(III), and four other commonly occurring metals—Na(I), K(I), Ca(II) and Fe (III)—are added with lead prior to adsorption experiments. It was observed that Pb(II) adsorption capacity of this carbon was not influenced by the presence of other metals. A highly elevated concentration of Na(I), K(I), Ca(II) and Fe(III) in the eluting solution compared to the initial dose suggested possible leaching of those metals from other salts as impurities, water source or even from the carbon itself, although the XPS analysis of the carbon confirmed negligible adsorption of those metals in carbon. From the equilibrium and kinetic data of adsorption, few parameters have been calculated, including distribution coefficient, diffusive time constant and pseudosecond order rate constant. The overall results suggest that these iron and sulfur dual-doped mesoporous carbons can serve as potential adsorbents for removal of lead from drinking water in the presence of other competing metals.

## 1. Introduction

Lead poisoning is one of the most common heavy metal poisonings and is quite prevalent in many areas of the world. Its origin can be traced back in wastewater and may be contributed to by various types of industries, including acid battery, glass manufacturing, printing, paints, oil, fertilizer, tanning and lead additives for gasoline [1,2,3,4]. In third world countries, drinking of poorly treated water also causes lead pollution. In western countries, the most common source of lead poisoning is through drinking water, which is caused by the leaching of lead from the older water pipes that contain lead among their constitutes. Lead is highly toxic and more prone to permanent damage or even death to children, young adults and pregnant women. It causes damage to the brain and central nervous system, impaired mental growth, kidney damage and reduction of sperm levels [5]. As lead is not an essential nutrient for mammals, it is mostly absorbed and stored in bones and the lead concentration in bones can go as high as 121 ppb. Besides humans or other mammals, lead is also poisonous to aquatic environment, like flora and fauna. Additionally, on an average basis, lead is also present in earth’s crust by 50 ppm and in sea water by 5 ppm [6]. According to Environmental protection agency (EPA) of USA, the lead level is drinking water should be less than 15 parts per billion (ppb or μg/L).

Additional purification of drinking water before consumption is always regarded as a safe practice to avoid lead intoxication. Different types of chemical and physiochemical separation techniques that have been employed to remove lead from water are chemical co-precipitation, electrochemical reduction, ion exchange, biosorption, reverse osmosis, membrane filtration and adsorption [7,8,9,10,11,12]. Adsorptive separation of lead by porous carbons has been regarded as the most popular choice for purifying drinking water, especially when the initial concentration of lead in water is in the lower range. Traditionally, activated porous carbons were synthesized from various types organic and agro-waste materials and have been employed for lead separation from water [13,14,15]. Despite activated carbon being the popular choice for removing lead, it has been realized that the general affinity of a heavy metal, like lead, towards pristine activated carbon, is not very high, leading to the poor adsorption capacity and selectivity. It was also demonstrated that inserting additional functionality on the surface of porous carbon can significantly enhance such affinity. In the literature, it was revealed that inserting sulfur functionalities on porous carbon increases its affinity towards heavy-metals [16,17], including lead [18,19] to a great extent. In fact, the toxicity of lead to the human body may be partly attributed to the affinity of lead towards sulfur component of protein where the lead can be irreversibly bound, thereby inhibiting the protein activity [20,21]. 

Despite the affinity of lead with sulfur-based sorbents being known, the competitive nature of lead in the presence of other metal cations is not well investigated and reported. In purification of drinking water, often other cations are mixed with lead and hence the nature of competition of metal ion adsorption needs to be investigated. Furthermore, as our study can be related to the environmental sustainability issues related to society, we believe that our manuscript is suitable to the scope of this journal. In order to pursue this work, we have synthesized soft-templated mesoporous carbons doped with both sulfur and iron (III) oxide functionalities. The soft-templated mesoporous carbon was synthesized with phloroglucinol as carbon precursor and pluronic F127 as porogen. The key reason for doping of sulfur and iron (III) oxide is that, while sulfur functionalities will enhance the affinity of lead onto the carbon surface, iron (III) oxide will make the carbon magnetic, thereby facilitating its separation from liquid medium by magnetic methods. In order to study the competitive adsorption, Cr(III) was used as competing heavy metal along few common metals that are also often present in the water, including Na(I), K (I), Ca(II) and Fe(III). In order to mimic the real-world situation for lead contaminated drinking water, the initial concentration of Pb(II) along with other metals is set as 100 parts per billion (ppb or μg/L). 

## 2. Experimental

### 2.1. Synthesis 

The first step of synthesis is to produce iron-doped mesoporous carbon by the soft-templating approach. The key synthesis step of fabricating mesoporous carbon is similar to that of our previously published method [22,23,24,25]. Typically, 5 g each of phloroglucinol (as carbon precursor) and pluronic F127 (as surfactant, [(PEO)_106_(PPO)_70_(PEO)_106_ M_w_ = 12,600; PEO: Polyethylene oxide, PPO: Polypropylene oxide) is dissolved in 25 mL of water and ethanol mixture (1:1 *v*/*v*) in the presence of 0.5 mL 36% HCl until they produce a transparent solution. After that, 2 g iron acetylacetonate (Fe(acac)_3_) was added in the mixture and continued to stir for an hour. Presence of Fe(acac)_3_ makes the color of the solution dark red. Then, 5 mL formaldehyde solution (37%) was added in the mixture. Formaldehyde cross-links phloroglucinol and a dark red colored gel is obtained after 45 min of stirring. The gel is separated from the reaction mixture and sprayed over a petri dish overnight. On the following day, the dried gel mass is broken into small fragments, loaded on a porcelain boat and inserted into the tube of a tube furnace (Lindburg-Blue^TM^) for carbonization. Typically, they are heated up to 400 °C at a ramp rate of 2 °C/min followed by 900 °C at the ramp rate of 5 °C/min and then cooled down to room temperature. All the heating and cooling operations are performed under N_2_ gas flow. Thus, obtained iron-doped mesoporous carbons were employed directly for sulfur doping without any modification. In order to synthesize pristine mesoporous carbon without any iron functionalities, the same protocol was pursued except the addition of iron acetylacetonate. 

**Figure 1:**
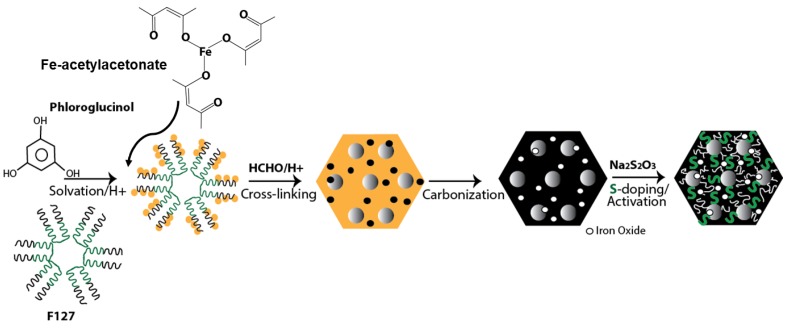
Schematic of synthesis of iron and sulfur dual-doped mesoporous carbon.

In order to introduce sulfur functionalities, iron-doped mesoporous carbons are mixed with solid and anhydrous sodium thiosulfate (Na_2_S_2_O_3_) within a coffee-grinder in the ratio of 1:1, 1:2 and 1:3. The solid mixture is loaded onto an alumina combustion boat and the boat is loaded within the same tube furnace. The furnace is heated up to 800 °C at the rate of 10 °C/min and then cooled down to room temperature. Like the previous method, the heating and cooling operations are performed under N_2_ gas. Upon cooling, the carbon is taken out from the furnace, washed with DI water several times, filtered and dried at 100 °C overnight. The iron and sulfur-dual doped carbons obtained with carbon to Na_2_S_2_O_3_ ratio of 1:1, 1:2 and 1:3 are named as MC-Fe-S1, MC-Fe-S2 and MC-Fe-S3, respectively. Pristine mesoporous carbon without any functionality is termed as MC. The schematic of overall synthesis method is shown in Figure 1.

### 2.2. Materials Characterization 

All the iron and sulfur dual-doped mesoporous carbons were characterized with surface functionalities with X-ray photoelectron spectroscopy (XPS) and pore textural properties, including BET surface area, micropore volume, mesopore volume and total pore volume. The XPS data were obtained in in a Thermo-Fisher K-alpha instrument with monochromatic Al-Kα as x-ray source. The energy of x-ray, instrument resolution, pass energy, step size and dwell time were 1486 eV, 0.5 eV, 50 eV, 0.1 eV and 50 ms, respectively. Each sample was mounted on a carbon tape followed by irradiation with 2 eV Ar^+^ ions to neutralize the charges. The pore textural properties were calculated by N_2_ adsorption at 77 K and CO_2_ adsorption at 273 K in Quantachrome’s Autosorb iQ surface area and porosity analyzer. BET surface area, mesopore volume and total pore volume were calculated based on N_2_ adsorption, whereas CO_2_ adsorption was employed to calculated micropore distribution and micropore volume. All the pore size data were calculated based on the Non-Local Density Function Theory (NLDFT) method. Scanning electron microscopy (SEM) and Energy dispersive X-ray (EDX) were performed in JEOL 7500F HRDEM for MC-Fe-S2 only as it demonstrated the highest lead adsorption and explained later. The magnetic properties of the carbons were measured by the Quantum Design’s physical property measurement system at the temperature 300 K and a magnetic field was applied up to 30,000 Oersted (Oe).

### 2.3. Adsorption Studies 

All the adsorption experiments were performed in batch mode in a round bottom flask with 25 mL solution and 0.025 g adsorbent (dual-doped mesoporous carbons) (concentration of adsorbent 1 g/L) under constant stirring. Metal nitrates, i.e., Pb(NO_3_)_2_, Cr(NO_3_)_3_, Fe(NO_3_)_3_, NaNO_3_, KNO_3_ and Ca(NO_3_)_2_ were used as the sources of the corresponding metals. In order to determine the best adsorbent, equilibrium studies were performed with the aqueous solution of 100 ppb pure Pb (II) for each of the mesoporous carbons (MC, MC-Fe-S1, MC-Fe-S-2 and MC-Fe-S-3) with pure Pb(II) only without any competing metals. Upon determining the best adsorbent, all the following equilibrium and kinetic studies were performed with best adsorbent only. For equilibrium adsorption studies of pure lead, the initial concentration was set to 20, 40, 60, 80 and 100 ppb and time interval of 3 h. For kinetic studies of pure Pb (II), initial concentration was set to 100 ppb and the time intervals were chosen as 2 min, 10 min, 30 min, 1 h, 2h and 3 h. One batch adsorption study was performed for each interval of time. For mixture (competitive) equilibrium adsorption, the initial concentration of each of Pb(II), Cr (III), Fe(III), Ca(II), Na(I) and K(I) was set to 20, 40, 60, 80 and 100 ppb along with constant stirring time of 3 h. In this experiment, the concentration of each element at any of concentration level was the same; for example, at 20 ppm concentration level, the initial concentrations for Pb(II), Cr (III), Fe(III), Ca(II), Na(I) and K(I) were all 20 ppm, i.e., total concentration 120 ppm. For kinetics of competitive adsorption, the same time interval as that of pure component was chosen along with an initial concentration of 100 ppb with respect to each of the metals. At the end of the adsorption studies, the aqueous solution was separated from the mesoporous carbons by simple filtration. The concentration of lead in pure component mode was determined by atomic absorption spectroscopy (AAS) (Shimadzu). The concentration of metals in competitive (mixture) mode was determined by Inductively coupled plasma (ICP) measurement (Thermoscientific ICP-MS). 

## 3. Results and Discussion

### 3.1. Materials Characteristics

**Figure 2:**
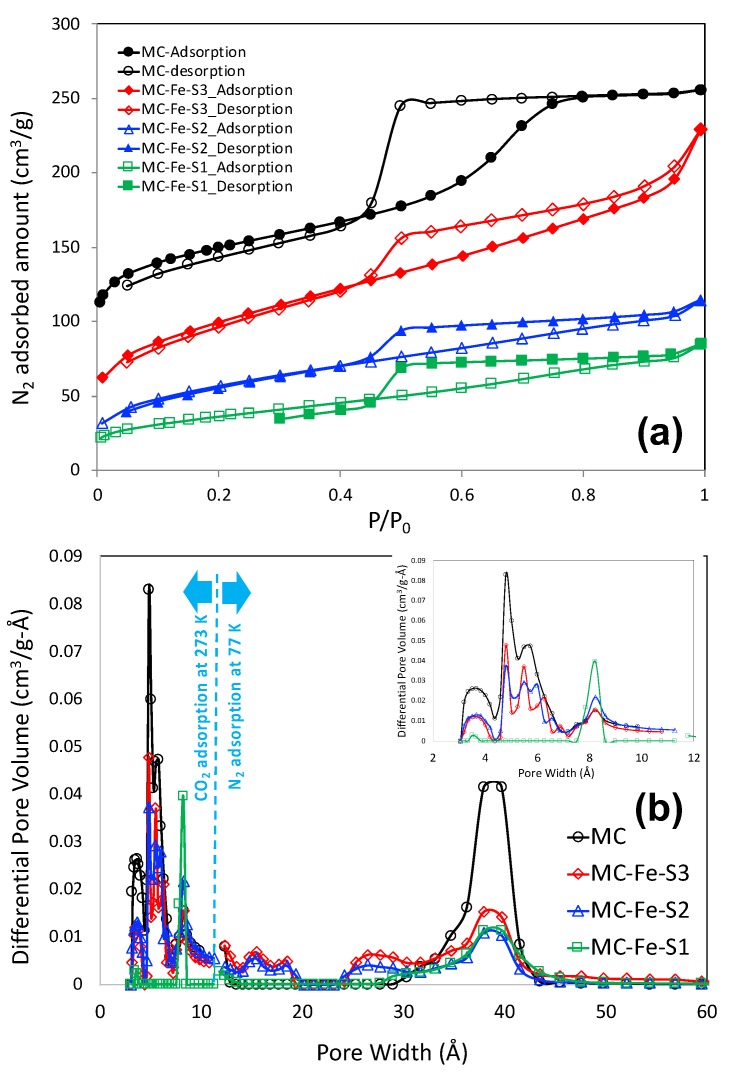
N_2_ adsorption–desorption plots at 77 K (**a**) and pore size distributions (**b**) of iron and sulfur dual-doped carbons. obtained by nonlocal density function theory (NLDFT). Inset of Figure 2b shows micropore distribution.

Nitrogen adsorption–desorption plot of all the mesoporous carbons at 77 K and pressure up to 1 bar is shown in Figure 2a. It is observed that all the isotherms are of type IV type according to IUPAC classifications. As observed in the isotherm, capillary condensation at P/P_0_ = 0.4–0.8 indicates mesopores in carbon material. The hysteresis loop at p/p_0_ at around 0.5 may also suggest that the carbon shell has a possible entrance pore. N_2_ adsorption–desorption isotherms at 77 K and CO_2_ adsorption isotherms at 273 K were employed to calculate the total pore size distribution, by nonlocal density function theory (NLDFT), whereas a CO_2_ adsorption isotherm is employed to calculate pore size distribution below 10 Å and a N_2_ adsorption isotherm was employed to calculate the same above 10 Å. The pore size distribution plots of all the mesoporous carbons are shown in Figure 2b and the corresponding pore textural properties are shown in Table 1. It is important to note that pristine mesoporous carbon (MC) possesses the highest BET surface area (509 m^2^/g) and total pore volume (0.363 cm^3^/g) including the individual micropore and mesopore contributions. Inclusion of Fe and S functionalities in the carbon surface lowers both BET surface area and pore volume. Interestingly, increase in sodium thiosulfate (Na_2_S_2_O_3_) content in the course of sulfur doping (from MC-Fe-S1- to -S3) also increases the overall porosity of the carbons that might have caused partial activation caused by Na_2_S_2_O_3_ itself. In addition to introducing sulfur functionalities, Na_2_S_2_O_3_ also partially reacted with the carbon matrix to create additional porosity in the system similar to that of traditional chemical activation caused by KOH and NaOH. As observed in Figure 2b, all the carbons have a discrete mesopore width of 40 Å. All the carbons also have distributed micropore widths centered around 4.79 and 5.73 Å. Fe and S dual-doped mesoporous carbons have micropore widths in the region of 8.21 Å that might have caused Na_2_S_2_O_3_, as mentioned earlier. Additionally, a very narrow micropore width is also observed at around 3.49 Å, which might be the graphitic layer spacing and not a true micropore.

**Figure 3:**
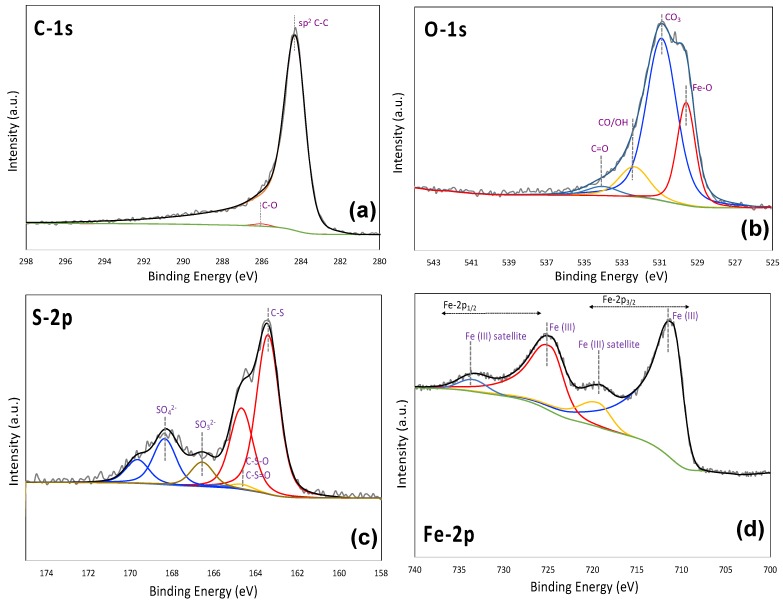
Representative XPS peak fitting of C-1s (**a**), O-1s (**b**), S-2p (**c**) and Fe-2p (**d**) spectra for MC-Fe-S2.

The atomic compositions mesoporous carbons are obtained by XPS and the corresponding C, O, Fe and S contents are shown in Table 2. One set of representative C1-s, O-1s, Fe-2p and S-2p peak fitting results for MC-Fe-S2 is also shown in Figure 3.

As observed in this table, the mesoporous carbons are mainly sp^2^ hybridized carbon with minor sp^3^ state as impurities. With the increase in Na_2_S_2_O_3_ content, total sulfur content increased from 4.4 to 6.1 atom% in MC-Fe-S1 to MC-Fe-S2, but further increasing in Na_2_S_2_O_3_ content decreased the total sulfur of MC-Fe-S3 to 5.9 atom%. The primary sulfur functionalities include C-S, C-S=O/C-S-O and SO_x_ type, where C-S functionalities are the dominant type. All the carbons also have about 18 to 24 atom% oxygen and the majority of them are attached to the carbon matrix in different forms of functionalities. The origin of those functionalities can be attributed to the oxygen content of carbon precursor (phloroglucinol) and reaction with Na_2_S_2_O_3_ in the course of sulfur functionalization. The total iron content of MC-Fe-S1,-2 and -3 is 8.6, 9.0 and 7.8 atom%, respectively. The majority of the iron functionalities are present in the form of ferric oxide (Fe_2_O_3_) along with small amounts of ferrous sulfate (FeSO_4_) (1.2 to 1.8%) that might have been formed due to the reaction between ferric oxide and sulfur functionalities in the course of sulfur doping. It also needs to be noted that MC-Fe-S2 has the largest amounts of both iron and sulfur in its matrix. It needs to be noted that despite sodium thiosulfate being used as a sulfur doping agent on the carbon, the dopant sulfur concentration on carbon may not directly be related to the amount of dopant or the stoichiometric ratio. Furthermore, sodium thiosulfate has both oxygen and sulfur in its structure and both of them can be incorporated onto the carbon matrix upon reaction. A closer inspection of Table 2 suggests that there is an increase in oxygen containing functionalities on the carbon surface (C=O, COOH, O-C=O) with the increase in sodium thiosulfate content (MC-Fe-S1: <0.1 at.%; MC-Fe-S2: 0.4 at.% and MC-Fe-S3: 2.5 at.%). Although the mechanism by which sodium thiosulfate reacts with carbon is unknown, most likely, a ratio of sodium thiosulfate may facilitate oxygen-containing functionalities over sulfur functionalities on the carbon surface. 

**Figure 4:**
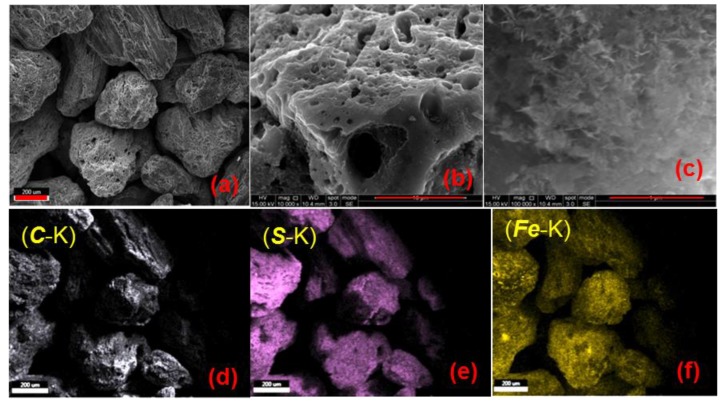
Scanning electron microscopic (SEM) images of MC-Fe-S2 in different magnifications ((**a**)–(**c**); (**a**) scale bar: 200 μm, (**b**) scale bar: 10 μm, (**c**) scale bar 1 μm). Energy dispersive X-ray image of MC-Fe-S2 for C-K (**d**), S-K (**e**) and Fe-K (**f**), all scale bars 200 μm.

The scanning electron microscopic (SEM) images and the corresponding energy dispersive x-ray (EDX) mappings of MC-Fe-S2 are shown in Figure 4. According to SEM image (Figure 4a), the average particle size is in the range of 400–600 μm. The surface of the particles has clearly visible larger macropores in the range of 1–5 μm (Figure 4b). Such hierarchical pore systems including the presence of macropore in addition to the micropore and mesopores may assist in the better diffusion and transport of adsorbing species, like metal ions. An SEM image with higher resolution (Figure 4c) also demonstrated a few needle-like crystal formations on the surface of the carbon matrix with a length of about 250 nm or less. Most likely, those crystals belong to iron oxides or iron sulfates. EDX mapping for C-*K*, S-*K* and Fe-*K* is shown in Figure 4d–f, respectively. According to the images, there is a homogeneous distribution of those elements in the system without any possible hotspot generation of a specific element.

**Figure 5:**
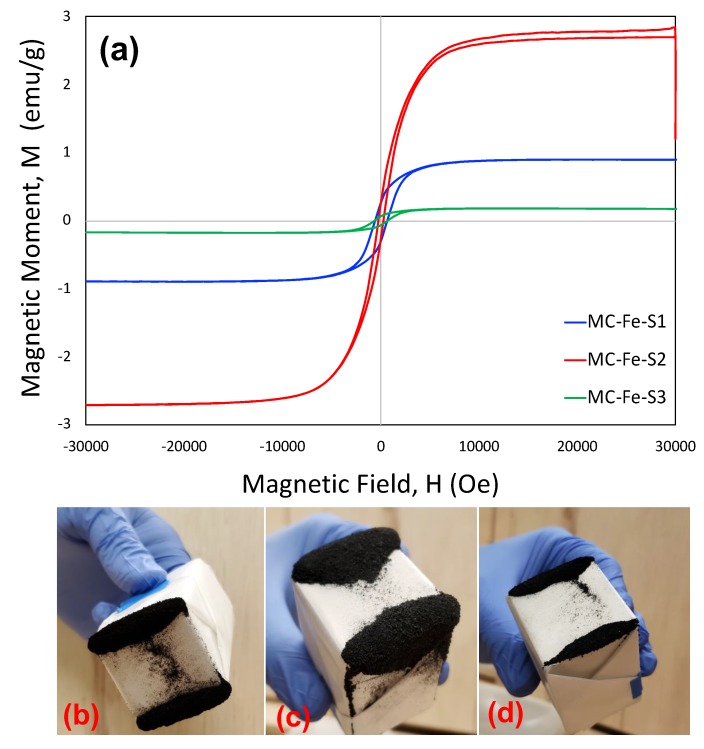
Demonstration of magnetic properties of iron and sulfur dual-doped mesoporous carbons by magnetization experiment (**a**), physical attractions of mesoporous carbons with neodymium magnet for MC-Fe-S1, (**b**) MC-Fe-S2, and (**c**) MC-Fe-S3 (**d**).

The magnetization plots of the three mesoporous carbons are shown in Figure 5a. It was observed that MC-Fe-S2 demonstrated the highest magnetic moment for a given magnetic field followed by MC-Fe-S1 and MC-Fe-S3. It is important to note that MC-Fe-S2 has the largest amount of iron or iron(III) oxide which is a key contributor of magnetic properties. The magnetic properties of the carbons are also demonstrated by a simple test with a permanent magnet. A neodymium magnet is wrapped with a tracing paper and brought in contact with the carbon powders. It was observed that all the carbons were attracted by the magnet and resultant snapshots are shown in Figure 5b–d for MC-Fe-S1, MC-Fe-S2 and MC-Fe-S3, respectively. It is also important to note that MC-Fe-S2 was able to attract the largest volume of carbon and hence supports the magnetization results. 

### 3.2. Adsorption Studies

**Figure 6:**
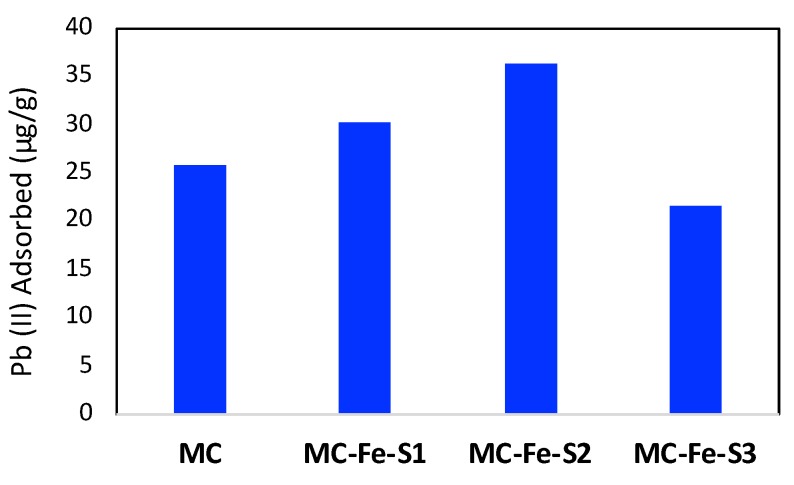
Pure Pb (II) adsorption data in pure mesoporous carbon (MC) and iron and sulfur dual-doped mesoporous carbons. All the experiments were performed in batch mode in 25 mL 100 ppb (μg/L) Pb(II) and 0.025 g adsorbent (Adsorbent concentration 1 g/L).

As the best adsorbent studies were made in one adsorption point only, the bar plot demonstrating the remaining concentration of Pb(II) after adsorption is shown in Figure 6. As observed in the figure, MC-Fe-S1 and MC-Fe-S2 performed better than pristine mesoporous carbon. Among all the carbons, MC-Fe-S2 appeared to be the best adsorbent.

It also needs to be noted that all the dual-doped mesoporous carbons have lower surface area and pore volume than pristine mesoporous carbon and hence superior lead adsorption must have been caused by the surface functionalities. It is also obvious that MC-Fe-S2 has the highest amount of sulfur that may facilitate the adsorption of Pb(II). A heavy-metal, like lead, has a high affinity towards sulfur functionalities, especially towards thiol groups on the carbon surface. It was also suggested that not all of Pb(NO_3_)_2_ may dissociate to form lead ion (Pb^2+^); it may stay an undissociated or partially dissociated species [26], like Pb(NO_3_)^+^. According to Pearson’s soft acid base theory (HSAB [27,28] hard acids can favorably bind with hard acids and soft acids with soft bases. Sulfur-doped carbons may act as soft base. Generally, neutral or partially ionized species are softer acids than completely ionized species and therefore partially dissociated un-dissociated lead salt may favor binding with sulfur functionalized mesoporous carbons. Additionally, metallic lead itself is considered as an intermediate acid with absolute hardness factor (η) to be [29]. 8.46 eV that may also assist in its adsorption in sulfur functionalized carbon. 

**Figure 7:**
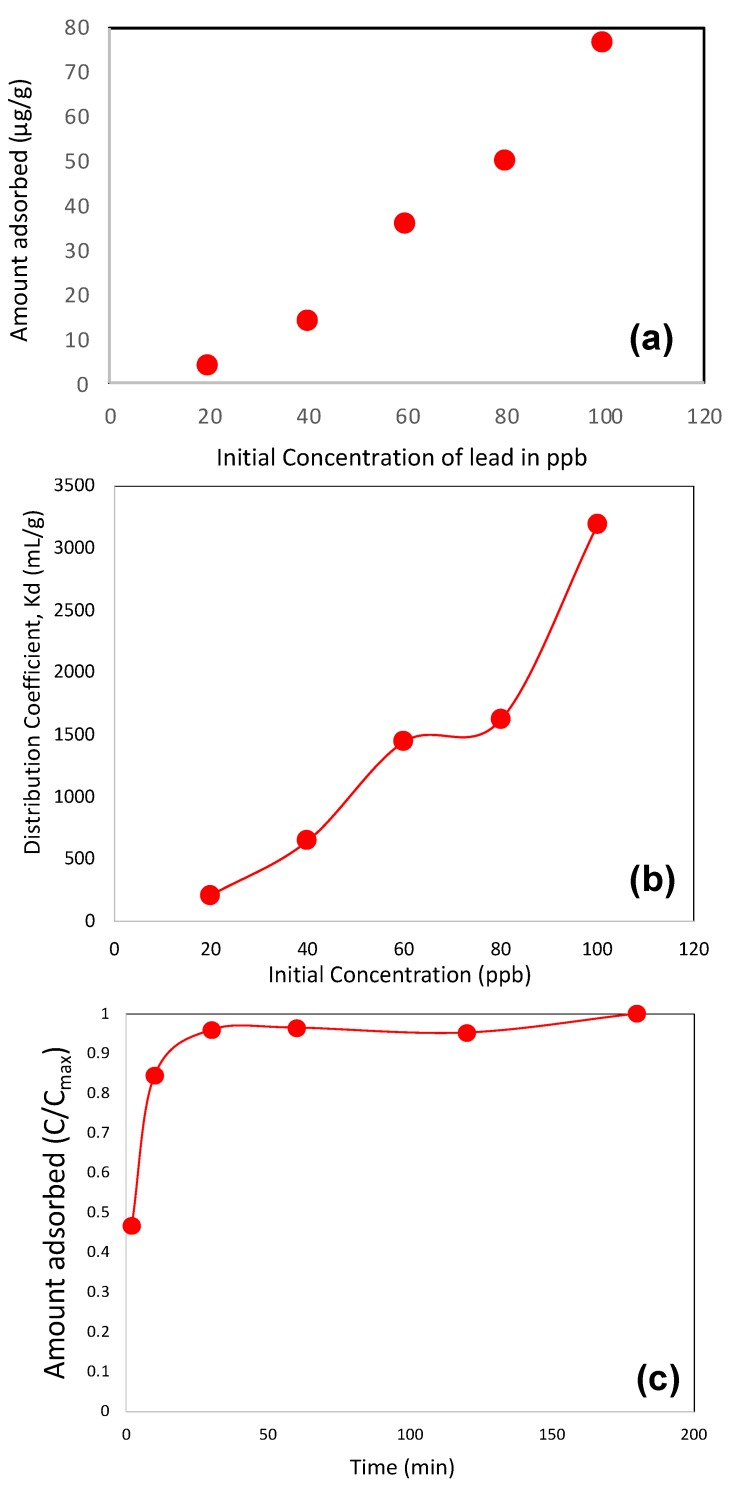
Pure Pb (II) adsorbed amount as a function of initial concentration; (**a**) distribution of coefficient (K_d_) of pure Pb(II) as a function of initial concentration (**b**) and adsorption kinetics of pure Pb(II) (**c**). The kinetic studies were performed in batch mode with Pb(II) concentration 100 ppb (μg/L) and for all experiments, the adsorbent concentration was 1 g/L.

Figure 7a shows the adsorbed amount of pure Pb(II) as a function of initial dose (concentration). As expected, the adsorbed amount increases with the increase in initial dose and the adsorbed amount is about 76 mg/g at the initial dose of 100 ppb. Pure Pb(II) equilibrium and kinetic adsorption data were also employed to calculate various adsorption parameters and the results are shown in Figure 7b,c, respectively. The distribution coefficient (*K_d_*, mL/g) is calculated as.

(1) $$K_{d}=\frac{C_{i}-C_{f}}{C_{f}}\left(\frac{V}{m}\right)$$

where *C_i_* is the initial concentration (ppb), *C_f_* is final concentration (ppb), *V* is adsorption mixture volume (mL) and *m* is the mass of adsorbent (g). As observed in Figure 7b, *K_d_* values of pure Pb(II) lie within 212 to 3196 mL/g and increase with the increase in the initial lead concentration. The kinetics of lead adsorption confirmed that the adsorbent reached its saturation level within 30 min of operation only.

In order to fit the kinetic data, the pseudosecond order rate equation is applied and it is given as [30].

(2) $$\frac{t}{q_{t}}=\frac{1}{k_{2}q_{e}^{2}}+\left(\frac{1}{q_{e}}\right)t$$

where *q_t_* is the equilibrium adsorbed amount at time *t*, *q_e_* is the equilibrium adsorbed amount and *k_2_* is the pseudosecond order rate constant. The rate constant can be calculated by linear regression of *t/q_t_* and *t* plot. The degree of fitting (R^2^) was within 0.95 to 0.99 and the second order rate constant of pure Pb(II) adsorption is given in Table 3. In order to find the diffusivity values, the micropore diffusion model was employed and given as [31].

(3) $$1-\frac{m_{t}}{m_{\infty }}=\frac{6}{\pi^{2}}exp\left(\frac{-\pi^{2}D_{c}t}{r_{c}^{2}}\right)$$

where $m_{t},$ $m_{\infty }$, *D_c_* and *r_c_^2^* are the adsorbed amounts at time *t,* equilibrium adsorbed amount, intracrystalline diffusivity and intracrystalline radius, respectively. For carbon-based materials, the intracrystalline radius is unknown and hence it is a common practice to represent the value of *D_c_*/*r_c_^2^*, which is referred to as diffusive time constant. The value of this diffusive time constant for pure Pb(II) adsorption is also given in Table 3. 

Part of the results of the competitive adsorption studies are counterintuitive. While measuring the concentration of the competitive metal ions after adsorption, we found that, except for Cr(III), the concentrations of Na(I), K(I), Ca(II) and Fe(III) are excessive, in the order of 200–2000 ppb and obviously, there is no trend in adsorption pattern. One set of representative concentrations of Na(I), K(I), Ca(II) and Fe (III) for equilibrium, kinetics and pH studies are shown in Table S1 of the supporting information. As these concentration values are somewhat higher than the initial concentration that we deliberately inserted in the system before adsorption (100 ppb), we investigated the other sources where those metals originated from. It was determined that Na(I) and K(I) were the largest and present in varying amounts of almost all the ingredients, that include the other metal nitrates and even in ultrapure water. Furthermore, we also suspect that those two metal ions can leach out from unlikely sources, like even glass utensils or filter paper. Owing to their omnipresence in almost all the ingredients, it was not possible to exclude them. Presence of Ca(II) was the smallest, and it was contributed to by other metal salts. We found that the key source where Fe(III) leached from is the porous carbon itself (as the carbons have iron as one of its constituents) and its concentration is in the range of 300–3000 ppm. As the metallic nitrate will generate partially acidic solution in water even without any pH adjustment, iron leaching could be partially facilitated by the acidic environment. Omnipresence of those metals also suggests that the apparent ‘pure’ Pb(II) studies, as we mentioned earlier, are not exactly pure, as few of the metals were always present. Furthermore, it was also possible to elevate the initial concentration of Pb (II) to minimize the relative presence of other metals, but we did not attempt to do it as that will no longer mimic the lead concentration in drinking water in a real-world scenario. In order to provide more insight and make an indirect study on the possibility of adsorption of other metals by the carbon, we have performed XPS analysis of the carbon upon completion of adsorption and explained this at the end of this section. 

**Figure 8:**
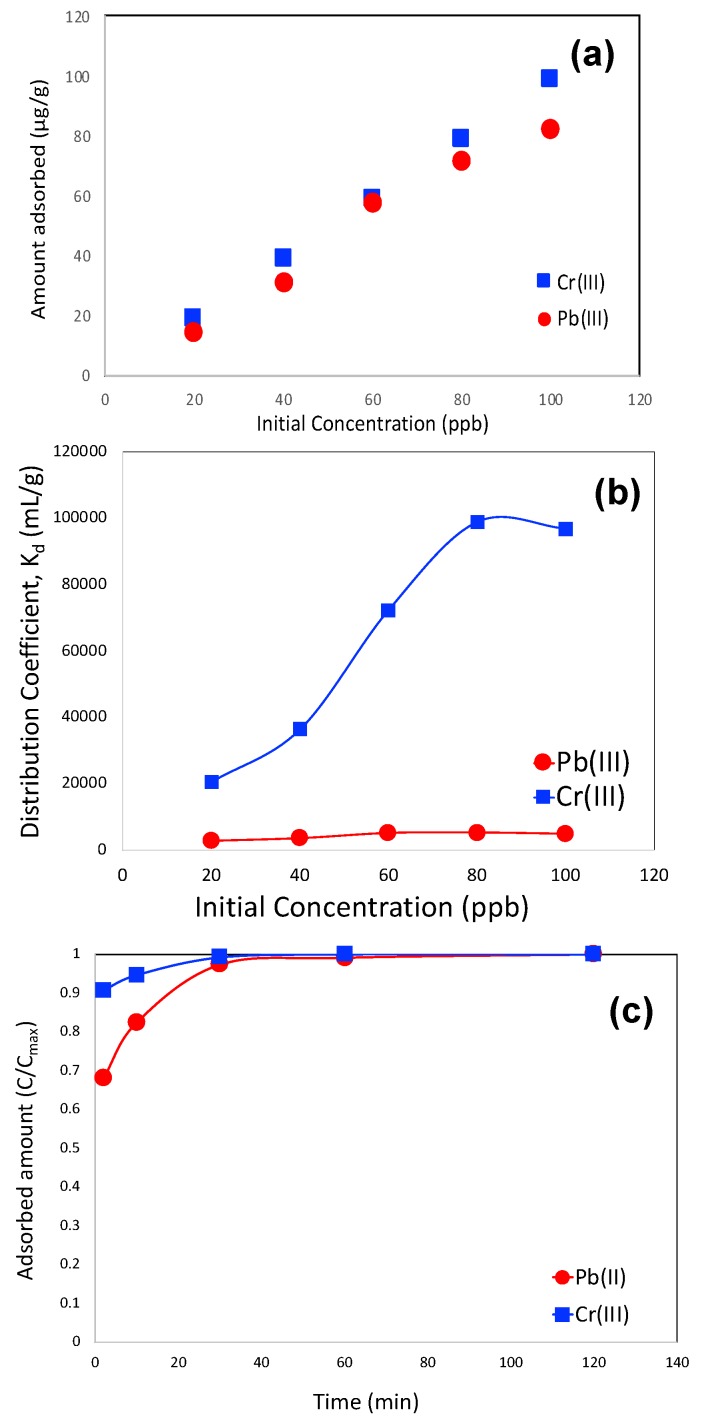
Pb (II) and Cr (III) adsorbed amount as a function of their initial concentration (**a**) Distribution coefficient (K_d_) of Pb(II) and Cr (III) in the mixture (**b**), Kinetics of Pb(II) and Cr(III) adsorption (**c**) The kinetic studies were performed in batch mode with Pb(II) and Cr(III) concentration 100 ppb (μg/L) and for all experiments, adsorbent concentration was 1 g/L.

The adsorbed amounts of Pb(II) and Cr(III) in the competitive mode are shown in Figure 8a. It was observed that the adsorbent is more selective to Cr(III) compared to Pb(II) as the Cr(III) adsorbed amount is slightly higher than Pb(II) adsorption, which is about 98 mg/g at the initial dose of 100 ppb. It is important to note that the Pb(II) adsorbed amount did not decrease compared to that of pure mode and hence most likely, Cr(III) did not compete with Pb(II) for the active sites, like sulfur functionalities. Furthermore, a very low concentration of the heavy metal did not block the pore space to lower the adsorption capacity for Pb(II). The distribution coefficient (K_d_) for Pb(II) and Cr(III) in the competitive adsorption mode are shown in Figure 8b. Owing to the higher adsorbed amount of Cr(III), the distribution coefficient of Cr(III) demonstrated higher values compared to Pb(II), as observed in this figure. It is interesting to note that, as mentioned in earlier discussion, chromium is considered as a hard base, so according to classical HSAB theory, the adsorption of Cr (III) is less favorable than Pb(II). Therefore, most likely, Cr(III) adsorption is favored by the classical dispersion forces owing to its higher charge. 

The kinetics of Pb(II) and Cr(III) adsorption are shown in Figure 8c. It was observed that Pb(II) adsorption was slightly more sluggish than that of Cr(III). Similar to that of pure Pb(II) adsorption, micropore diffusion and pseudosecond order rate models were applied to both Pb(II) and Cr(III) adsorption kinetics data and diffusion time constant and pseudosecond order rate constants were calculated. Those values are shown in Table 3 along with pure Pb(II) adsorption data. In this regard, it is worth mentioning that we also tried to fit a pseudofirst order rate equation, but it did not fit and therefore, we did not report it. 

As observed in the table, there is no significant change in diffusion time constants and pseudosecond order rate constants for competitive and non-competitive Pb(II) adsorption. Only a small difference that is registered in those constants may be attributed to the difference in degree of fitting. It suggests that Pb(II) adsorption was mostly unaffected by the presence of other commonly used metal ions in water if their concentration is low enough. The mechanism of adsorption of the metals in porous carbons consists of four stages, (i) transport of metal species from the bulk of the solution to the proximity of the carbon, (ii) transport of the species through the boundary layer on the surface of the carbon (film diffusion), (iii) transport of the species from the surface to the pores of the carbon (intraparticle diffusion), and (iv) adsorption or chemical complexation of the species at the pores or active sites. The intraparticle diffusion is governed by the equation,

(4) $$q_{t}=K_{id}t^{1/2}+C$$

where *q_t_* is the adsorbed amount at time *t* and *K_id_* is the intraparticle diffusion constant (mg g^−1^ min^−1/2^ [32]. The process of adsorption is limited by the intraparticle diffusion if a linear regression plot of *q_t_* versus *t* passes through the origin. In our calculation, we did not find such a trend and hence intraparticle diffusion was not a controlling factor for the system. 

**Figure 9:**
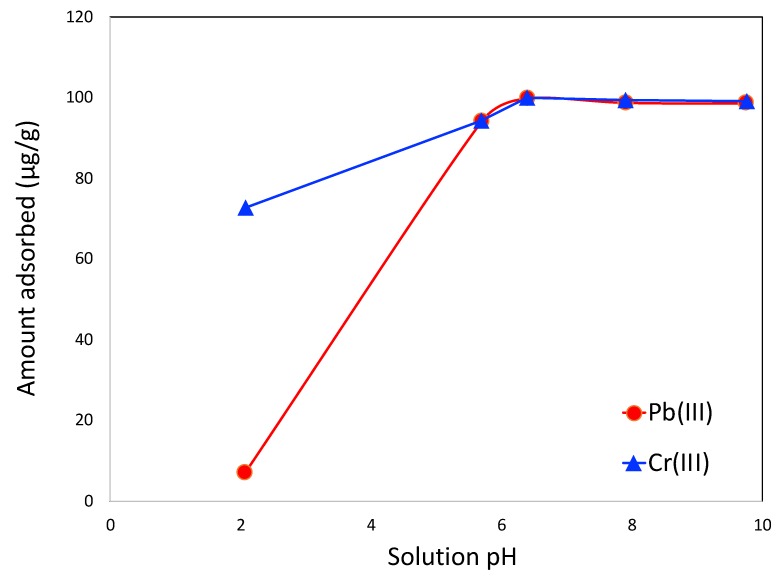
Influence of solution pH in the adsorption of Pb(II) and Cr (III) in mixture. The pH studies were performed in batch mode with a Pb(II) concentration of 100 ppb (μg/L) and the adsorbent concentration was 1 g/L.

The pH dependency of Pb(II) adsorption in the competitive mode is shown in Figure 9. It is observed that the adsorbed amount is lower in the lower pH of the mixture. It increases at the neutral pH and continues to achieve the same level of adsorption at elevated pH or basic medium. It is also noticeable that Cr(III) adsorption maintains a similar trend. Such pH dependency of Pb(II) is also observed in the previous reports on lead adsorption in different types of porous carbons. At lower pH, carbon surface is positively charged. This charge may cause electrostatic repulsion between positively charged lead or partially dissociated lead ions and lowers the adsorbed amount. At neutral pH, the carbon surface is no longer positively charged and hence adsorption is improved. At higher pH, the carbon surface is negatively charged, and it should further improve the adsorption of positively charged species, like lead ions. However, at higher pH, the presence of additional hydroxyl ions (OH^−^) causes increased competition for adsorption between hydroxyl ions and lead species and that may limit the adsorption of lead onto the carbon surface.

In order to further understand the fate of adsorbed ions onto the carbon surface, we performed XPS analysis of the carbons upon completion of adsorption studies in competitive mode. We detected a very small amount of lead and calcium on the carbon surface, but could not detect other metals, except iron. Pb(II) has a higher cross-section of x-ray compared to Cr(III) and that is probably the reason for not detecting Cr(III), despite its adsorption amount being slightly higher. Quite interestingly, two of the other metals—Na(I) and K(I)—were not detected despite their concentration being higher (some cases, even an order of magnitude higher) than Pb(II) in the residual solution upon adsorption, as mentioned earlier. XPS only detected a small amount of Ca (II) (0.1 at.%). The XPS peak fitting results for Pb and Ca are shown in the supporting information (fig S1 and S2). Such incidence suggests that adsorption of those metals is probably negligible in the mesoporous carbons. No definite conclusion could be made on iron adsorption by XPS as iron was always present on the carbon surface as part of its functionalities. 

## 4. Conclusions

In this research, iron and sulfur dual-doped mesoporous carbons were successfully synthesized. The carbons possessed a BET surface area of 130–352 m^2^/g, total pore volume 0.11–0.3 cm^3^/g, sulfur content 4.4–6.1 atom % and iron content 7.8–9 atom%. One heavy metal, Cr(III), and a few other metals, including Na(I), K (I), Ca(II) and Fe(III), were used as competitive metals in the course of Pb(II) adsorption. In order to mimic the real-world condition of drinking water, the concentration (dose) of Pb(II) and other metals was kept in the range of 100 ppb. Despite Cr(III) adsorption being slightly higher than Pb(II), it was observed that the Pb(II) adsorbed amount was not influenced in competitive mode compared to non-competitive mode, suggesting the successful role of the adsorbent in the real-world use. It was revealed that the concentrations of Na(I), K(I), Ca (II) and Fe(III) were higher after adsorption, which may be caused by the impurities in other salts, DI water, utensils or even leaching from the carbon itself. XPS of the carbonaceous adsorbent after adsorption could not detect Na(I) and K(I), thereby suggesting negligible adsorption of those metals by the adsorbent. The overall results suggest that iron and sulfur dual-doped mesoporous carbons can be employed as a potential adsorbent to remove lead from drinking water.

## Figures and Tables

**Table 1:** Pore textural properties of mesoporous carbons.

Adsorbents	BET SSA (m^2^/g)	Micropore Volume (cm^3^/g)	Mesopore Volume (cm^3^/g)	Total Pore Volume (cm^3^/g)
MC	509	0.147	0.216	0.363
MC-Fe-S1	130	0.0019	0.115	0.117
MC-Fe-S2	205	0.0046	0.152	0.157
MC-Fe-S3	352	0.0083	0.298	0.306

**Table 2:** Atomic contents of the mesoporous carbons, as obtained from XPS.

MC-Fe-S1				MC-Fe-S2				MC-Fe-S3			
C	63.0%	C-C sp^2^	57.1%	C	60.5%	C-C sp^2^	59.7%	C	67.6%	C-C sp^2^	53.0%
		C-C sp^3^	2.6%			C-C sp^3^	<0.1%			C-C sp^3^	10.2%
		C-O	3.2%			C-O	0.4%			C-O	1.9%
		C=O COOH O-C=O	<0.1%			C=O COOH O-C=O	0.4%			C=O COOH O-C=O	2.5%
S	4.4%	Fe-S	<0.1%	S	6.1%	Fe-S	<0.1%	S	5.9%	Fe-S	<0.1%
		C-S	2.6%			C-S	4.1%			C-S	4.0%
		C-S-O C-S=O	0.4%			C-S-O C-S=O	0.1%			C-S-O C-S=O	0.7%
		-SO_x_	1.4%			-SO_x_	1.8%			-SO_x_	1.2%
O	24.1%	Fe-O	5.2%	O	24.4%	Fe-O	5.8%	O	18.7%	Fe-O	5.3%
		Other	18.9%			Other	18.6%			Other	13.4%
Fe	8.6%	Fe_2_O_3_	~5.2%	Fe	9.0%	Fe_2_O_3_	~5.8%	Fe	7.8%	Fe_2_O_3_	~5.3%
		FeSO_4_	~1.4%			FeSO_4_	~1.8%			FeSO_4_	~1.2%

**Table 3:** Kinetic model fitting parameters for adsorption studies.

Metals	Diffusion Time Constant (D_c_/r_c_^2^) (s^−1^)	Pseudosecond Order Rate Constant (k_2_) (g^−1^mg^−1^s^−1^)
Pb (II), pure	1.52 × 10^-4^	8.83 × 10^−5^
Pb (II), mix	1.01 × 10^-4^	1.18 × 10^−4^
Cr (III), mix	1.54 × 10^-4^	4.70 × 10^−4^

## Supplementary Materials

The following are available online, Figure S1: XPS Pb-4f peak deconvolution results of MC-Fe-S2 after competitive adsorption, Figure S2: XPS Ca-2p peak deconvolution results of MC-Fe-S2 after competitive adsorption Table S1: Representative Concentrations of Na(I), K(I), Ca(II) and Fe(III) in kinetic, equilibrium and pH studies
