# Supplementary material for: Soft-Templating of Sulfur and Iron Dual-Doped Mesoporous Carbons: Lead Adsorption in Mixtures
Source: Molecules. 2020 Jan 18;25(2):403. doi: 10.3390/molecules25020403 (PMC7024228; doi:10.3390/molecules25020403)
Supplement: Supplementary file 1 [file molecules-25-00403-s001.pdf]

Table S1. Representative Concentrations of Na(I), K(I), Ca(II) and Fe(III) in kinetic, equilibrium and pH studies

**Type of study**	Na(I) (ppb)	K(I) (ppb)	Ca(II) (ppb)	Fe (III) (ppb)
Equilibrium studies: Initial Concentration:100 ppb	2056.564	1804.56	1014.03	306.29
Kinetic studies: 2 hour study	1378.76	1108.34	892.63	331.88
pH studies: 2.07	1785.78	1679.34	1599.10	3092.04

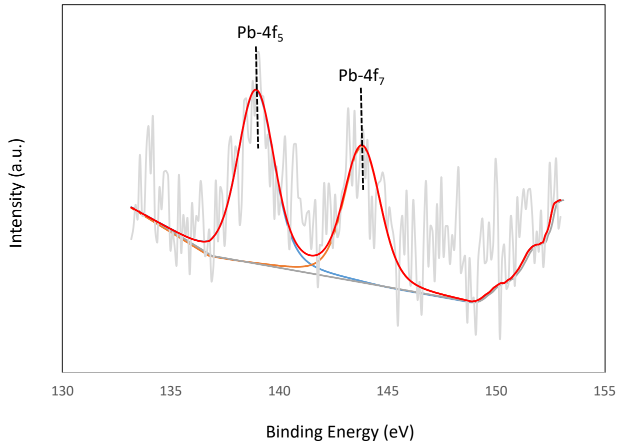

Figure S1. XPS Pb-4f peak deconvolution results of MC-Fe-S2 after competitive adsorption

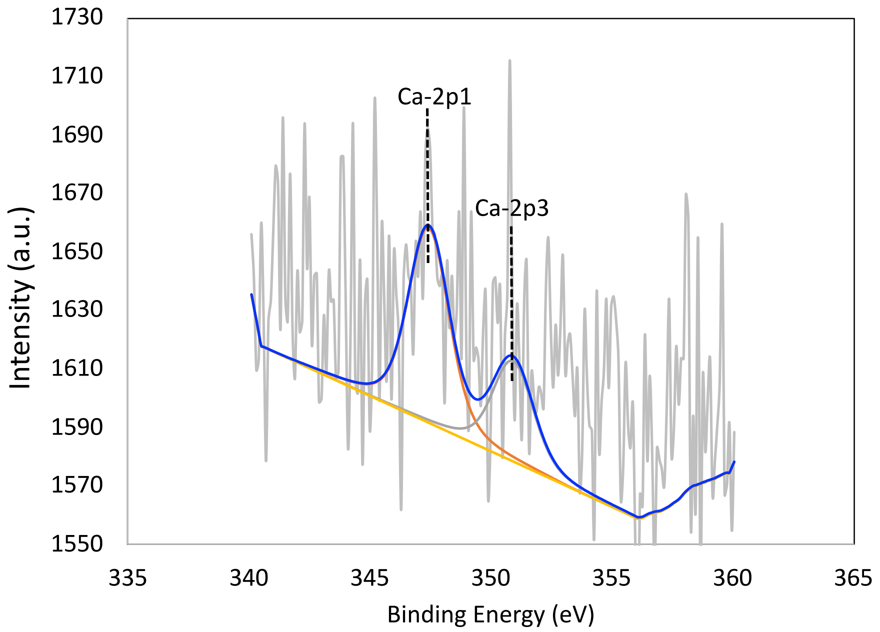

Figure S2. XPS Ca-2p peak deconvolution results of MC-Fe-S2 after competitive adsorption
